# Field Evaluation of a Ready-to-Use Porcine Circovirus Type 2 and *Mycoplasma hyopneumoniae* Vaccine in Naturally Infected Farms in Taiwan

**DOI:** 10.3390/vetsci12040304

**Published:** 2025-03-26

**Authors:** Fu-Chun Hsueh, Chia-Yi Chien, Shu-Wei Chang, Bo-Rong Lian, Hong-Yao Lin, Leonardo Ellerma, Ming-Tang Chiou, Chao-Nan Lin

**Affiliations:** 1 Department of Veterinary Medicine, College of Veterinary Medicine, National Pingtung University of Science and Technology, Pingtung 91201, Taiwan; jim820723@gmail.com (F.-C.H.); a8033376159483@gmail.com (C.-Y.C.); 2 Intervet Animal Health Taiwan Ltd., Taipei 11047, Taiwan; hikouki767777@gmail.com (S.-W.C.); b10216026@gmail.com (B.-R.L.); 3 MSD Animal Health Innovation Pte Ltd., Singapore 718847, Singapore; hongyao.lin@msd.com; 4 MSD Animal Health (Phils.), Inc., Makati City 1226, Philippines; leonardo.ellerma@merck.com; 5 Animal Disease Diagnostic Center, College of Veterinary Medicine, National Pingtung University of Science and Technology, Pingtung 91201, Taiwan; 6 Research and Technical Center for Sustainable and Intelligent Swine Production, National Pingtung University of Science and Technology, Pingtung 91201, Taiwan

**Keywords:** *Mycoplasma hyopneumoniae*, porcine circovirus type 2, porcine respiratory disease complex, vaccination, swine, bivalent vaccine, *Actinobacillus pleuropneumoniae*

## Abstract

Porcine circovirus type 2 (PCV2) and *Mycoplasma hyopneumoniae* (MHP) are both important and common pathogens in the pig industry. Both pathogens are major contributors to the porcine respiratory disease complex and serve to potentiate other bacterial infections such as *Actinobacillus pleuropneumonia*. This study aims to evaluate the efficacy of a ready-to-use bivalent PCV2 and MHP vaccine in the field under naturally PCV2-infected farms against existing monovalent options. We evaluated PCV2 viremia, PCV2 antibodies, and lung lesion scores in slaughtered pigs in our study across four farms in Taiwan. Our results found that in two out of four farms, the piglets vaccinated with Porcilis^®^ PCV M Hyo had superior whole-life PCV2 viremia reduction compared to the existing vaccination program on farms. In the lung lesion scoring, the Porcilis^®^ PCV M Hyo group had significantly lower *Actinobacillus pleuropneumonia*-type lesions in pigs than in the competitor group in two out of three farms evaluated. In this field trial, Porcilis^®^ PCV M Hyo proved to be efficacious in protecting piglets against both PCV2 viremia and the impact of MHP secondary infection, in the context of a reduction in viremia and reduced APP-like lesions found at slaughter.

## Simple Summary

Porcine circovirus type 2 (PCV2) and *Mycoplasma hyopneumoniae* are common swine pathogens that cause respiratory disease and economic losses in the pig industry. We vaccinated pigs with a vaccine (Porcilis^®^ PCV M Hyo) against these two pathogens in four naturally infected farms and compared the performance against the existing measures already in use on the farm. Several parameters were used to compare vaccine efficacy: antibody and viremia levels of PCV2 and lung lesion scoring at slaughter for *Mycoplasma hyopneumoniae.* We found that antibody levels and viremia were improved in the majority of farms. In terms of lung lesion scoring, we found that the levels of secondary bacterial infection associated with *Mycoplasma hyopneumoniae* were reduced. In conclusion, vaccination with Porcilis^®^ PCV M Hyo improved control of PCV2 and *Mycoplasma hyopneumoniae* on farms.

## 1. Introduction

The term “porcine respiratory disease complex” (PRDC) refers to a multifactorial disease that arises from a combination of viral, bacterial, and, less frequently, parasite, environmental, managerial, and genetic factors, leading to the development of pneumonia in pigs [1]. A primary pathogen involved in PRDC is porcine circovirus type 2 [2,3] (PCV2). PCV2 has been widely studied over the last few years and is the main pathogen responsible for porcine circovirus-associated diseases (PCVADs), which are characterized as clinical or subclinical PCV2 infections among pigs [2,3]. In the early 2000s, PCV2 vaccination was rare and the most common presentation was postweaning multisystemic wasting syndrome (PMWS) [4]. This is clinically characterized by wasting, respiratory disease and enteritis [4]. After twenty years of vaccination, field infection pressure has reduced and today the common presentation seen in the field is subclinical disease, characterized by decreased average daily gain (ADG) and increased feed conversion ratio (FCR) [5]. These diseases are associated with enormous economic losses in industrial pork production. For instance, Alarcon et al. [6] estimated the costs of PCVAD in the UK as ranging from GPB 84.1 to GBP 8.1 per pig, depending on the severity of the infection. In the United States, the disease has cost producers an average of USD 3–4 per pig with peak losses in severe infection up to USD 20 per pig [7]. From an immunological perspective, PCV2 targets lymphoid tissues and strongly impacts T-cell selection processes in the thymus [2,8], resulting in lymphoid depletion and, consequently, immunosuppression in the pig. Consequently, PCV2 infection has been associated with reduced vaccine responses after vaccination [9] and increased severity of other swine pathogens such as *Actinobacillus pleuropneumoniae* (APP), pseudorabies virus (PRV) and porcine reproductive and respiratory syndrome virus (PRRSV) [10].

Another primary agent of PRDC is *Mycoplasma hyopneumoniae* (MHP). MHP is also the etiological agent of porcine enzootic pneumonia (EP), a chronic respiratory disease that affects pigs [11]. MHP infections are endemic in swine farms around the world and have major economic impacts due to costs of treatment and vaccination, decreased feed conversion rate, and increased mortality resulting from secondary infections [12]. The economic impact per finisher pig was estimated at EUR 2–6 by Ferraz et al. [13]. MHP infection leads to epithelial damage of the swine respiratory tract, resulting in ciliostasis and eventual cell death. The loss of cilia predisposes the host to additional secondary infection from pathogens such as APP, *Streptococcus suis* or *Glaesseralla parasuis* (GPS) [14].

APP is the etiologic agent of porcine pleuropneumonia, a highly contagious pulmonary disease in pigs. A common presentation of APP in the field is acute mortality in finishing pigs. The death of finisher pigs represents a significant economic loss to producers due to the sunk costs of raising pigs to slaughter age [15]. The severity of APP lesions is worsened in the presence of co-infection with PCV2 and MHP [16]. In the absence of co-infections, infection with APP can be subclinical to asymptomatic [17].

To evaluate the efficacy of control measures on farm, veterinarians have several methods to assess the level of infection of PCV2, MHP and APP. For PCV2, the most important presentation in the field is subclinical, with associated economic losses due to slowed or uneven growth rates in a batch of pigs. Increases in viremia levels have been correlated with decreases in zootechnical parameters [18,19], making viremia an important metric to assess the efficacy of PCV2 infection control measures. Engle et al. [18] found that PCV2 viraemia was an important driver of ADG decline following infection; a moderate negative correlation was observed between viral load and overall ADG. For MHP and APP, visual inspection of lung lesions in the slaughterhouse is a reliable way to assess the overall disease status of the herd [20]. EP-like lesions scored at slaughter have been positively associated with the herd MHP seroprevalence at market weight [21]. The magnitude of cranioventral pulmonary consolidation lesions on lungs is correlated with an ADG decline [13]. APP has been isolated via bacterial culture from caudal lung lesions observed in conjunction with pleurisy and hemorrhagic abscesses [22]. The visual observation of these lesions thus serves as a proxy for understanding the prevalence of APP in a herd.

At present, producers in Taiwan have multiple commercial vaccines available for the control of PCV2 and MHP. These vaccines are administered to sows or piglets and at different times in the life cycle of the pig. Despite this, both diseases continue to exhibit a high prevalence in commercial swine [23,24,25] and new solutions are needed. At present, a novel ready-to-use (RTU) vaccine is available on the market in Taiwan (Porcilis PCV M Hyo). Multiple field and laboratory studies have already demonstrated the efficacy and safety of this product in Europe [26,27,28,29,30]. The objectives of our study were to evaluate the efficacy of this vaccine in field conditions in Taiwan through comparative trials based on whole-life PCV2 viremia and lung lesion evaluation in slaughterhouses.

## 2. Materials and Methods

### 2.1. Ethical Statement for Experimental Procedures

The animal experimental procedure was reviewed and approved by the Institutional Animal Care and Use Committee (IACUC) of the National Pingtung University of Science and Technology (NPUST), with approval number NPUST-109-009.

### 2.2. Herd Information and Experimental Design

Four commercial swine farms located in Taiwan were enrolled in the study (Table 1). Herd identification was performed in cooperation with swine veterinarians to find herds which had been vaccinating with PCV2 and MHP vaccines for at least 5 years but still reported incidence of respiratory disease in either the nursery or finishers. The herd swine veterinarians also reported, in all four farms, the incidence of dry coughing in the finisher stage, which is pathognomonic for MHP infection. No GPS vaccine was in use on all four farms. All herds were positive for PCV2 and MHP and, to a varying degree, other respiratory pathogens such as PRRS and APP. During the study period, all farms were classified as positive stable for PRRSV and monitored by the Animal Disease Diagnostic Center, NPUST.

Vaccination strategies in the different herds prior to initiating Porcilis^®^ PCV M Hyo vaccination differed substantially (Table 1). Porcilis^®^ PCV M Hyo (MSD Animal Health, Rahway, NJ, USA) is a commercial ready-to-use bivalent vaccine against both PCV2 and MHP. This vaccine was first registered in the European Union in 2015 [31]. Piglets from a weaned batch were randomly divided into two groups; the first group used the current farm vaccination program and the second group was given Porcilis^®^ PCV M Hyo (MSD Animal Health, Rahway, NJ, USA) at 3 weeks of age (Table 1). Blood samples were randomly collected from 10 pigs per group at 3 weeks (before vaccination), 8 weeks, 12 weeks, 16 weeks, 20 weeks and 24 weeks of age, and the blood was sent to the Animal Disease Diagnosis Center of the College of Veterinary Medicine, National Pingtung University of Science and Technology. The serum was carefully transferred into 1.5 mL centrifuge tubes after centrifugation of blood samples at 2150× *g* for 15 min. In total, 120 blood samples were collected from each pig farm.

### 2.3. PCV2 Antibody and Viremia Testing

All serum samples were tested for the presence of PCV2 IgG antibody using a commercially available indirect ELISA kit (Porcine Circovirus type 2 Antibody Test, BioChek B.V., Reeuwijk, Holland) according to the manufacturer’s instructions. PCV2 virus nucleic acid detection from serum samples were performed by real-time PCR, which was developed by our laboratory according to the methods of Tsai et al. [23]. PCV2 loads of less than 10^3^, 10^3^ to 10^4^, and greater than 10^4^ DNA copies/μL of serum sample can be used to categorize pigs as subclinically infected, suspected, and PCVAD, respectively [32,33,34]. PCV2 load dynamics were also analyzed using the area under the curve (AUC) as an indicator the PCV2 load over the duration of this study [35].

### 2.4. Lung Lesion Scoring

Lung lesion scoring (LLS) was conducted in Farms B, C and D. Here, only a portion of slaughtered pigs were evaluated due to the need to make special arrangements for pig slaughter to allow inspection at the slaughterhouse. The scoring method was based on the method proposed by Madec et al. [36]. The scoring criteria was set as 0 points for no consolidation on the lung lobes; 1 point for 1–25% coverage of lesions; 2 points for 26–50% lesion coverage; 3 points for 51–75% lesion coverage; and 4 points for lesions greater than 75%. The lesion scoring was carried out separately for the seven separate lung lobes, giving each pig a lung lesion score ranging from 0 to 28 points.

### 2.5. Statistical Analysis

Fisher’s exact test was used to compare the positive rate, and Student’s *t*-test was used to determine the viral load of each group. The Kruskal–Wallis test was used to compare the AUC between the groups. The lesion index of pigs in slaughterhouses in each group was determined by a chi-square test. *p* values < 0.05, <0.01 and 0.001 were considered statistically significant, highly significant and very highly significant, respectively.

## 3. Results

### 3.1. Comparison of PCV2 Antibodies

**Figure 1:**
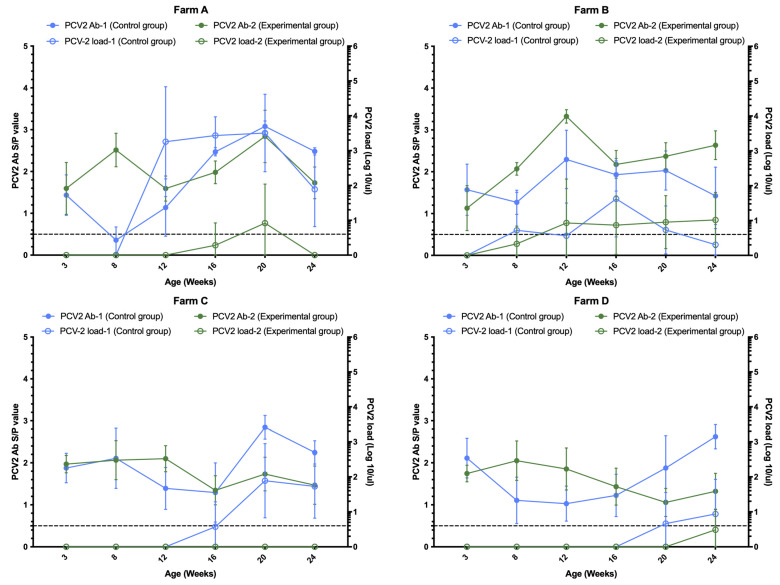
Comparison of PCV2 antibody and viremia at different weeks of age in Farms A, B, C and D. Blue and green lines represent control and experimental groups (Porcilis^®^ PCV M Hyo), respectively. Solid and hollow represent PCV2 antibodies and viremia at different weeks of age. The dotted line indicates the threshold for the presence of PCV2 antibody (0.5). The X-axis represents different ages. The left Y-axis represents the S/P value of PCV2 antibody. The Y axis on the right represents the PCV2 viral load. Error bars indicate 95% confidence interval for the mean.

The results show that there was no significant difference between control and experimental groups before vaccination (at 3 weeks of age), but at 8 weeks of age there was a positive trend in the experimental group (group 2) in Farms A, B and D (Figure 1), while the control group (group 1) only had a slight increase in PCV2 antibody in Farm C (Figure 1), and the other three farms (A, B and D) showed a downward trend (Figure 1). The peak antibody rate in the Porcilis^®^ PCV M Hyo vaccine group (experimental group) was found at 8–12 weeks; then, the antibody count decreased, and the PCV2 antibody count increased slightly again around 20 weeks. However, in the control group, significant positive conversion of PCV2 antibody was detected at 16 weeks in Farms A, C and D (Figure 1).

### 3.2. Comparison of PCV2 Viral Load and Positivity Rate

**Figure 2:**
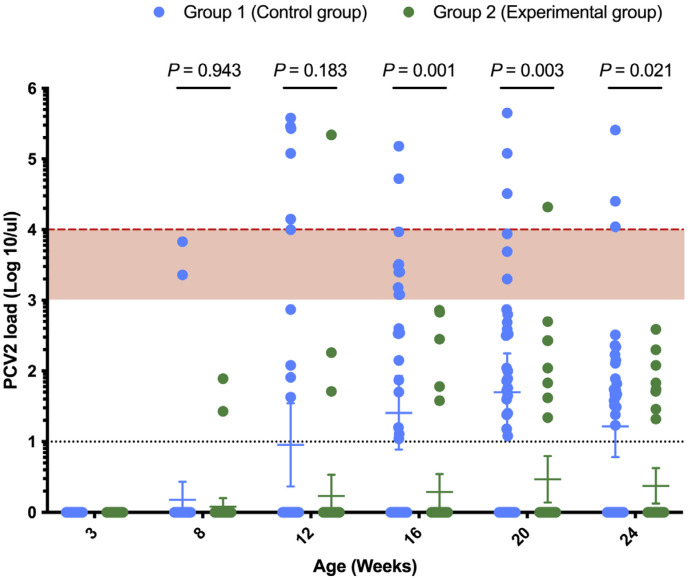
Comparison of PCV2 viremia between the two groups at different ages in the study. Blue and green represent group 1 (control group) and group 2 (experimental group), respectively. PCV2 loads of less than 10^3^, 10^3^ to 10^4^ (red area), and greater than 10^4^ DNA copies/μL (red dotted line) in serum can be used to categorize pigs as subclinically infected, suspected, and PCVAD, respectively. The black dotted line is the PCV2 qPCR detection limit, and the blue and green on the X axis represent a negative PCV2 qPCR assay. Error bars indicate 95% confidence interval for the mean. *p* values < 0.05, <0.01 and 0.001 are considered statistically significant, highly significant and very highly significant, respectively.

Across all four farms, PCV2 infection occurred around the 16–20-week age mark, as indicated by the increase in antibody titers in that age group across both groups 1 and 2 (Figure 1). The viremia of group 2 in each farm was lower than that of group 1, with the most obvious difference in the performance of Farm A (Figure 1). Figure 2 summarizes the overall PCV2 viral load in different age groups in this study, and the results show that at 16 weeks, 20 weeks and 24 weeks of age, the viral load of the experimental group (group 2) was significantly lower than that of the control group (group 1). The PCV2 viral load was greater than 10^4^ genome copies/μL in more pigs in group 1 compared to group 2 (Figure 2). There was a very highly significant difference between the two groups in Farm A and Farm C (Table 2) in terms of AUC of PCV2 viremia. We found no differences in the positive rates of PCV2 viremia between the two groups before or 5 weeks after vaccination, but from 12 weeks of age to 24 weeks of age, the positive rates of PCV2 viremia in group 1 were significantly higher than those in group 2, with statistically significant differences (Table 3).

### 3.3. Lung Lesion Scoring

Our results found that the lung lesion index was different among different farms (Table 4), and there was no significant difference in the mean lung lesion index between the two groups in Farms B, C or D (Table 4). However, the proportion of lungs without any lesions in all farms was the highest in group 2 (group 2: 8.09% vs. group 1: 15.06%). The average lung lesion score was numerically superior in group 2 (group 1: 2.52 vs. group 2: 1.4). Across all farms, group 2 pigs had a higher proportion of pigs with lung lesions below4 points (Table 4—96.7% in Farm B, 96.9% in Farm C, 91% in Farm D) compared to group 1.

### 3.4. Comparison of Other Relevant Lesions in Slaughterhouses

We found that there were significantly fewer pulmonary lesions associated with *Actinobacillus pleuropneumonia* in Farm C and Farm D when lung lesions were observed in the slaughterhouse (Table 5). We also observed that the incidence of pleuritis in Farm C’s experimental group was significantly lower than that in the control group (Table 5). In addition, three pigs in Farm D’s group 1 had lungs affected by severe pleurisy and were not able to be scored based on lung lesions. This was presumed to be caused by severe GPS or APP infection (Table 4).

## 4. Discussion

The objective of our study was to evaluate the field efficacy of an RTU bivalent PCV2 and MHP vaccine. The previous vaccination program on all four farms consisted of monovalent PCV2 and MHP vaccines. A bivalent RTU vaccine would thus have advantages over normal monovalent vaccines by reducing the number of injections needed to administer two antigens, bringing biosecurity, animal welfare and worker efficiency advantages. In terms of biosecurity, swine vaccines are commonly administered through intramuscular (IM) injection [37] via sterile needles. Needle reuse is common [38] and raises the possibility of iatrogenic transfer of disease between animals. An RTU vaccine that reduces injections would reduce the need to reuse needles in swine. PRRSV has been shown to spread to susceptible pigs via contaminated needles [39,40]. The same has been demonstrated for African swine fever virus [41] and PCV2 [42]. From a welfare perspective, the study of Owen et al. [38] found substantial damage to the needle after 12 uses, leading to increased skin shearing requiring higher force to puncture the skin and consequentially additional local trauma and inflammation post vaccination. This potentially contributes to both swine welfare and meat quality issues, particularly if needles are reused [43,44]. From a worker efficiency perspective, labor costs are increasing in many farms in Asia and labor cost is an increasing issue for many farms [45]. The time saved from reducing the need to pick up pigs twice translates into a reduction in manhours spent vaccinating pigs.

In our study, we found that pigs in the control group presented an elevated PCV2 viremia between 16 and 20 weeks of age, coupled with increases in PCV2 antibody levels, indicating a field challenge. Similarly to a previous study [25], seroconversion was observed in the fattening stage of pigs in all herds in this study. Upon slaughter, more than 74% presented with MHP-like lung lesions, with average LLS of 1.3 to 4.8. These elements confirm that both PRDC pathogens were actively circulating in the trial farms despite regular on-going use of vaccination over the previous five years or longer and justify the trialing of a new vaccine to establish if better control of PCV2 and MHP could be obtained.

In terms of efficacy comparison, a whole-life PCV2 viremia reduction compared to existing control options was observed in two out of four farms in all groups vaccinated with Porcilis^®^ PCV M Hyo. Interestingly, despite higher antibody titers against PCV2 in group 1 of Farms A, C and D, higher overall levels of viremia were recorded in the same groups. Group 2 in these farms had lower overall viremia despite lower titers. The commercial ELISA we used is not able distinguish antibodies induced by vaccination or field challenge [46], and a higher level of antibodies may be indicative of higher levels of viral infection. Viremia reduction is a key metric used to assess PCV2 vaccine efficacy, and pigs with higher levels of viremia have a higher risk of developing PCVAD [46]. We demonstrated PCV2 viremia compared with the existing product in use on farms (Farms A, C and D). Despite not weighing pigs, other authors have published work correlating reductions in PCV2 viremia with improvements in ADG [19]. We therefore speculate that a reduced PCV2 viremia would translate into an improved ADG. Compared to the control group, the experimental group vaccinated with Porcilis^®^ PCV M Hyo had numerically similar or better outcomes regarding the frequency of lungs without pneumonia and average lung lesion score. Our results also found that the incidence of APP-like lesions was significantly reduced in two out of three farms. The presence of PCV2 and MHP co-infection has been shown to potentiate APP [10,16,47]. Other authors have also demonstrated that improving control of MHP on farm results in a reduction in APP-like lesions observed at slaughter [48]. We thus hypothesize that as the farms were all not using APP vaccines or engaging in antimicrobial prophylaxis for APP, the improvements seen were due to improved control of PCV2 and MHP.

This study has at least two limitations. We were not able to weigh the pigs for the calculation of ADG due to farm labor restrictions and we were limited by the numbers of animals we were able to follow to slaughter to conduct lung lesion scoring. In Taiwan, pigs are sold through an auction system [49], and after the auction, they will be separated from their original consignment and hence not easily traceable at the slaughterhouse. We therefore had to arrange for pigs to be slaughtered in a single consignment to allow for lung lesion scoring and tracking. The lowered numbers of lungs observed possibly contributed to the lack of significant differences observed in lung lesion scores at the slaughterhouse. Despite this, we were able to observe significant differences in APP occurrence, suggesting that reductions in MHP impact were indirectly observed.

## 5. Conclusions

In this field trial, Porcilis^®^ PCV M Hyo proved to be efficacious in protecting piglets against both PCV2 viremia and the impact of MHP secondary infection, in the context of a reduction in viremia and reduced APP-like lesions found at slaughter.

## Figures and Tables

**Table 1:** Basic information of the experimental pig herd, immunization schedule and vaccine brand.

Farm	Area	Number of Sows	Vaccination Program for PCV2 and *Mycoplasma hyopneumoniae* (MHP) in Sows	Vaccination Program for PCV2 and MHP in Piglets	Vaccine Brand
A	Tainan	750	No immunity	1. PCV2:10 days old/MHP: 10 and 24 days of age 2. PCV2: 3 weeks old/MHP: 3 weeks of age	1. PCV2-1/MHP-2 2. Porcilis^®^ PCV M Hyo
B	Yunlin	1550	No immunity	1. PCV2: 3 weeks old/MHP: 3 days old 2. PCV2: 3 weeks old/MHP: 3 weeks of age	1. PCV2-1/MHP-2 2. Porcilis^®^ PCV M Hyo
C	Chiayi	3000	PCV2: 5 weeks before delivery	1. PCV2 + MHP: 21–24 days old 2. PCV2: 3 weeks old/MHP: 3 weeks of age	1. PCV2-1/MHP-1 2. Porcilis^®^ PCV M Hyo
D	Changhua	650	PCV2 and MHP at weaning	1. PCV2: 3 weeks old/MHP: 3 weeks old 2. PCV2: 3 weeks old/MHP: 3 weeks of age	1. PCV2-1/MHP-3 2. Porcilis^®^ PCV M Hyo

**Table 2:** Comparison of the area under the curve of PCV2 viremia.

Farm	Group ^a^	Area Under the Curve	*p* Value ^b^
A	1	445.58	0.0001
	2	48.04	
B	1	155.06	0.988
	2	145.33	
C	1	132.98	0.0001
	2	0	
D	1	45.28	0.054
	2	9.72	

^a^ 1: other vaccine brands; 2: Porcilis^®^ PCV M Hyo. ^b^ *p* values < 0.05, <0.01 and 0.001 are considered statistically significant, highly significant and very highly significant, respectively.

**Table 3:** Comparison of PCV2 viremia positivity rates among different ages.

Weeks of Age	N	Group (%) ^a^		*p* Value ^b^
		1	2	
3	40	0 (0)	0 (0)	-
8	40	2 (5)	2 (5)	1
12	40	10 (25)	3 (7.5)	0.0338
16	40	20 (50)	5 (12.5)	0.0002
20	40	25 (62.5)	8 (20)	0.0001
24	40	22 (55)	8 (20)	0.0012

^a^ 1: other vaccine brands; 2: Porcilis^®^ PCV M Hyo. ^b^ *p* values < 0.05, <0.01 and 0.001 are considered statistically significant, highly significant and very highly significant, respectively.

**Table 4:** Comparison of lung lesion index of pigs in slaughterhouse.

Farm	Group (n) ^a^	Lung Lesion Score					No. of Undetermined ^b^
		0 (%)	1–4 (%)	5–9 (%)	>10 (%)	Mean ± SD	
B	1 (30)	8 (26.7)	21 (70.0)	1 (3.3)	0 (0)	1.30 ± 1.21	0
	2 (30)	11 (36.7)	18 (60.0)	1 (3.3)	0 (0)	1.30 ± 1.42	0
C	1 (26)	4 (15.4)	10 (38.5)	10 (38.5)	2 (7.7)	4.38 ± 3.35	0
	2 (65)	11 (16.9)	52 (80.0)	1 (1.5)	1 (1.5)	1.94 ± 2.22	0
D	1 (11)	2 (25.0)	5 (62.5)	1 (12.5)	0 (0)	1.88 ± 2.64	3
	2 (11)	5 (45.5)	5 (45.5)	1 (9.1)	0 (0)	1.00 ± 1.48	0

^a^ 1: other vaccine brands; 2: Porcilis^®^ PCV M Hyo. ^b^ Due to severe pleurisy and damage of the lung, it was not possible to conduct lung scoring.

**Table 5:** Comparison of other related lesions in slaughtered pigs.

Farm	Group (n) ^a^	Pleuritis	Pericarditis	APP
B	1 (30)	1 (3.3)	0 (0)	0 (0)
	2 (30)	3 (10.0)	0 (0)	0 (0)
C	1 (26)	9 (34.6) **^b^	0 (0)	14 (53.8) **
	2 (65)	4 (6.2) **	3 (4.6)	0 (0) **
D	1 (11)	4 (36.4)	0 (0)	9 (81.8) **
	2 (11)	0 (0)	0 (0)	2 (18.2) **

^a^ 1: other vaccine brands; 2: Porcilis^®^ PCV M Hyo. ^b^ ** Highly significant difference between the two groups by chi-square analysis.

## Data Availability

The data presented in this study are contained within this article.
